# Long-distance dependency combined multi-hop graph neural networks for protein–protein interactions prediction

**DOI:** 10.1186/s12859-022-05062-6

**Published:** 2022-12-05

**Authors:** Wen Zhong, Changxiang He, Chen Xiao, Yuru Liu, Xiaofei Qin, Zhensheng Yu

**Affiliations:** 1 College of Science, University of Shanghai for Science and Technology, Jungong Road, Shanghai, 200093 China; 2 School of Optical-Electrical and Computer Engineering, University of Shanghai for Science and Technology, Jungong Road, Shanghai, 200093 China

**Keywords:** Multi-head self-attention, Long-distance dependency, Receptive field, Protein–protein interactions

## Abstract

**Background:**

Protein–protein interactions are widespread in biological systems and play an important role in cell biology. Since traditional laboratory-based methods have some drawbacks, such as time-consuming, money-consuming, etc., a large number of methods based on deep learning have emerged. However, these methods do not take into account the long-distance dependency information between each two amino acids in sequence. In addition, most existing models based on graph neural networks only aggregate the first-order neighbors in protein–protein interaction (PPI) network. Although multi-order neighbor information can be aggregated by increasing the number of layers of neural network, it is easy to cause over-fitting. So, it is necessary to design a network that can capture long distance dependency information between amino acids in the sequence and can directly capture multi-order neighbor information in protein–protein interaction network.

**Results:**

In this study, we propose a multi-hop neural network (LDMGNN) model combining long distance dependency information to predict the multi-label protein–protein interactions. In the LDMGNN model, we design the protein amino acid sequence encoding (PAASE) module with the multi-head self-attention Transformer block to extract the features of amino acid sequences by calculating the interdependence between every two amino acids. And expand the receptive field in space by constructing a two-hop protein–protein interaction (THPPI) network. We combine PPI network and THPPI network with amino acid sequence features respectively, then input them into two identical GIN blocks at the same time to obtain two embeddings. Next, the two embeddings are fused and input to the classifier for predict multi-label protein–protein interactions. Compared with other state-of-the-art methods, LDMGNN shows the best performance on both the SHS27K and SHS148k datasets. Ablation experiments show that the PAASE module and the construction of THPPI network are feasible and effective.

**Conclusions:**

In general terms, our proposed LDMGNN model has achieved satisfactory results in the prediction of multi-label protein–protein interactions.

## Background

Protein takes one of the most common molecules in organisms. It is the material basis of life activities and participates in various biological processes in organisms [1]. Most vital biological processes in organisms are generally driven by protein–protein interactions (PPIs), rather than an individual protein acting alone [2–4]. PPIs are widespread and play an important role in biological systems. For instance, PPIs are essential for biological cell activities such as cell proliferation, immune response, signal transduction, DNA transcription, and replication [5]. Therefore, exploring the interactions between protein and protein is the key to study cell biology [6–8] and has great significance to the diagnosis and treatment of diseases, as well as the design and development of drugs [9]. At present, there are many methods for the prediction of PPIs, which can be broadly divided into two types: laboratory-based traditional methods and deep learning methods.

In pace with the rapid development of high-throughput technology, a number of laboratory-based traditional methods have been used to predict PPIs, such as mass spectrometric protein complex identification (MS-PCI) [10], yeast two-hybrid (Y2H) [11, 12] and tandem affinity purification (TAP) [13, 14]. These methods can visually observe the interactions between protein and protein. However, the vast majority of experiments are based on genome scale, and has narrow comprehensive. At the same time, a lot of time and money are needed to support the smooth running of the experiment. In addition, part of experiments rely on obtaining target proteins from animals, which violates ethics and morality [15–19]. To address the shortcomings of traditional methods, researchers were turning to deep learning methods.

Deep learning, by virtue of its powerful feature learning ability, has been valued by various fields and is rapidly evolving, no exception in the bioinformatics field, where it is ingeniously applied to probe some problems in bioinformatics, such as protein–protein interactions prediction tasks. Sun et al. [20] applied stacked autoencoder (SAE) to capture amino acid sequence features to predict PPI. Hang et al. [21] designed a deep neural network (DNN-PPI) framework capable of automatically acquiring features in the amino acid sequence of proteins. Chen et al. [7] constructed an end-to-end framework for PPI prediction based on siamese residual recurrent neural network (PIPR), which extracted features from amino acid sequences. These deep learning models all exhibit excellent generalization ability for addressing the PPIs prediction tasks. However, they are highly dependent on the amino acid sequence of proteins. Since there may be some similarities between different amino acids, these typical deep learning models cannot effectively capture the information of the entire protein amino acid sequence and the relationships between different amino acids.

It should be noted that all the methods mentioned above only take amino acid sequence features as input. They don’t consider the interactions between proteins [22], which makes the prediction performance limited. Protein–protein interactions can be considered as hidden information to some extent, so combining it and amino acid sequence information together can improve the accuracy of prediction. For the PPI network, it can be viewed as a graph with each protein as the node and the connecting relationships between proteins as the edge.

Therefore, Yang et al. [23] proposed to view the PPI network as an undirected graph and applied GCN [24] in the PPIs prediction task for the first time. It constructed an unsigned variational graph autoencoder, which combined the PPI network with amino acid sequence information to learn the features of proteins to predict PPI. Inspired by the methods of graph signal processing, Colonnese et al. [25] considered node features on PPI networks as signals, and developed a Markov model to accomplish PPIs prediction. Lv et al. [26] constructed a GNN-PPI model based on graph isomorphism network (GIN) to predict the interactions between protein–protein pairs. It not only considered the amino acid sequence information, but also fully considered the correlation between proteins.

These models have improved the accuracy of PPIs prediction. However, they only considered the information of two proteins directly interacting in PPI network, ignoring the information of protein–protein pairs indirectly interacting. In this way, the information captured by these model are incomplete. Studies [27, 28] have shown that indirectly interacting also have meaningful information. In a biological network, two molecular nodes that do not interact directly also have some similarities [29]. And then, for the target node, it would be helpful if the information of such indirectly interacting nodes could be aggregated.

In this paper, we propose a novel LDMGNN model to implement the multi-label prediction task of protein–protein interactions. This model mainly aims to solve the two problems mentioned above. The first problem is that the existing methods ignore the long-distance dependence information between amino acids in the sequence, and the second is that the existing methods do not fully consider the interaction among indirect connected protein nodes. To solve the first problem, we use the Transformer with a multi-head self-attention mechanism to capture the correlation between every two amino acids in the sequence. For the second problem, we construct a two-hop protein–protein interaction (THPPI) network based on the PPI network to enhance graph representation learning. Overall, our main contributions:

- We use the Transformer module with a multi-head self-attention mechanism to capture the long-distance dependency information between each two amino acids in sequence.
- Based on the two-hop concept, we construct a THPPI network based on PPI network, which can capture the information between indirectly interacting proteins and thus increase the receptive field in space.
- The experimental results show that our method exhibit good performance.

## Resuts

In the following, the datasets, experimental parameter settings, evaluation metrics, baselines, experimental results and analysis used in our experiment will be introduced.

### Datasets

In this paper, we use two common datasets, i.e., SHS27k and SHS148k, to evaluate our method. These two datasets contain a lot of PPIs information and amino acid sequence information. They were randomly selected by [7] from the Homo sapiens subset of STRING [30] according to the rule that the sequence alignment similarity is less than $40 \%$. The SHS27k dataset contains 1690 proteins and 7624 pairs of PPIs, and the SHS148k dataset contains 5189 proteins and 44,488 pairs of PPIs. The interactions of the two datasets can be divided into seven types, i.e., posttranslational modification (ptmod), catalysis, reaction, activation, expression, binding and inhibition, they can show not only the physical correlation between proteins but also the functional correlation.

We regard all known PPIs as positive samples, and negative samples of the same size are randomly selected from unknown interactions. In our experiment, the positive and negative sample rate is 1:1. Specifically, in the SHS27k dataset, the number of negative samples is 7624. In the SHS148k dataset, the number of negative samples is 44,488. At the same time, inspired by [26], in order to evaluate the generalization ability of the LDMGNN model more realistically, we choose three partition schemes to divide the test set, i.e., random, BFS and DFS. Our test set accounts for $20 \%$ of the dataset.

Given the protein set *P* and PPI set *I* , construct a protein–protein interaction network $G=\langle {P}, {I}\rangle$. The size of the fixed test set is *N* (We divide the data set according to edges, that is, *N* refers to the number of edges in the test set). Firstly, a protein is randomly selected from the protein set *P* as the root node $p_{root}$. Given a threshold *t*, the degree of the root node $p_{root}$ must be less than this threshold (we set the threshold $t=5$), that is $\left| N\left( p_{\text{ root } }\right) \right| <t$. Set the initial test set $I_{\text{ test }}=\emptyset$, the current node $p_{cur} = p_{root}$. And then use DFS(BFS) algorithm to search the neighbor nodes $p_{k}$ of the current node $p_{cur}$, i.e. $p_{k} \in N\left( p_{cur}\right)$. At this time, the test set $I_{\text{ test }}=I_{\text{ test }} \cup I_{cur}$, $I_{cur}=\left\{ p_{cur}, p_{k}\right\}$. The process is repeated until the number of edges in the subgraph formed by all nodes in the test set exceeds *N*.

### Parameter settings and evaluation metric

Our experiment is performed on an NVIDIA GTX 3090 GPU with a PyTorch deep learning framework. We choose the Adam algorithm [31] as the optimization strategy in this paper with a weight decay coefficient of 5e−4 and a batch size setting of 512. We train our models for 300 epochs with an initial learning rate of 0.001. We choose the ReduceLROnPlateau function to vary the learning rate and to prevent overfitting, the patience is set to 20. During model training, if the loss is not reduced for 20 consecutive iterations, training will automatically stop.

Since our task is to use a classifier to solve the multi-label PPI classification. The interactions between protein–protein pairs have at least one label. Moreover, the types of PPIs in the SHS27k and SHS148k datasets are extremely unbalanced [26]. *Micro*-*F*1 will emphasize the common labels in the datasets, which is not easy to be affected by small samples or large samples, so that each sample has the same importance [32]. Comprehensive consideration, we choose the *Micro*-*F*1 evaluation metric to measure the accuracy of our model. The mathematical formula is as follows:

1 $$\begin{aligned} \ Micro{-}F1 = 2 \frac{Recall_m \times Precision_m}{Recall_m + Precision_m}, \end{aligned}$$

where $Recall_m$ and $Precision_m$ are the total recall and total precision for all classes, expressed with mathematical formulae as follows:

2 $$\begin{aligned} Recall_{m} = \frac{TP_{1}+TP_{2}+\cdots +TP_{n}}{TP_{1}+TP_{2}+\cdots +TP_{n}+FN_{1}+FN_{2}+\cdots +FN_{n}}, \end{aligned}$$

3 $$\begin{aligned} Precision_m = \frac{TP_1+TP_2+\cdots +TP_n}{TP_1+TP_2+\cdots +TP_n+FP_1+FP_2+\cdots +FP_n}, \end{aligned}$$

where *n* indicates the number of classes, in this experiment, the number of classes is 7. $TP_{i}$, $FP_{i}$, $TN_{i}$ and $FN_{i}$ indicate true positives, false positives, true negatives and false negatives of the *i*th class, respectively.

### Baselines

In order to better illustrate the effectiveness of our model, we compare LDMGNN with different baselines. These baselines can be divided into machine learning based and deep learning based. We choose three algorithms based on machine learning, which are SVM [33], RF [34] and LR [35]. The input are the features of proteins, which are common handcrafted protein features, i.e., AC [33] and CTD [36].

When compare with the models based on deep learning, we construct the same architecture as them. We input the SHS27k dataset and SHS148k dataset into the model, and change the output from the original two classification to multi-label classification. These deep learning models are as follows.

- *HIN2Vec* [37]: A representation learning framework for heterogeneous information networks (HIN). It uses different types of interactions among nodes to capture the features of nodes and meta- paths in HIN.
- *SDNE* [38]: A structural deep network embedding method for link prediction and multi-label classification tasks. It can not only effectively capture the highly nonlinear network structure, but also preserve the global and local structure of the network.
- *LPI-DLDN* [39]: A deep learning model of dual-network neural architecture composed of feature importance ranking (FIR) network and MLP network. Given the sequences of protein and lncRNA, predict the potential interaction between lncRNA and protein.
- *LPI-deepGBDT* [40]: A multiple-layer deep structure model based on gradient boosting decision trees. Given the sequences of protein and lncRNA, predict the unobserved LPIs.
- *DTI-CDF* [41]:A cascade deep forest model based on hybrid feature, which cascades the traditional machine learning models RF and XGB. Given the hybrid feature (contains the information of drug, target and drug-target interaction) to predict the interaction between drug and target.
- *PIPR* [7]: An end-to-end network model for predicting PPI, which combines two residual RCNN using Siamese architecture. And this method provides an automatic multi-granularity feature selection mechanism to capture the features of sequences.
- *GAT* [42]: A new neural network based on graph structure data. It learns the embedding of nodes by using self-attention mechanism in the structure of graph.
- *GNN-PPI* [26]: A graph neural network model, given the information of protein amino acid sequence and PPI network, is used for the prediction of multi-label PPI.

Our experiment is inspired by GNN-PPI [26]. However, compared with GNN-PPI model, our LDMGNN model mainly has the following two innovations. First, in the part of amino acid sequence encoding, we innovatively propose to replace biGRU block with a transformer block with multi-head self-attention mechanism, which can not only capture the long-distance dependence information between amino acids, but also solve the problem that biGRU cannot be parallelized. Second, considering that there may be some connection between nodes that do not interact directly, in order to capture more comprehensive information of proteins, we construct a two-hop PPI network. This is not available in the GNN-PPI model.

### Results and analysis

As shown in Table 1, the LDMGNN method shows the best performance compared to the other baselines. From this result, it can be seen that our model has fully learn the long-distance dependency between amino acids and effectively expand the receptive field, which can improve the prediction accuracy of multi-label PPI. From the perspective of dataset size, the performance of the model increases with the size of the dataset. Obviously, our method performs better under dataset SHS148k than dataset SHS27k, this is because we are able to obtain more valuable information as the PPI network growing. From the perspective of dataset partition scheme, the performance improvement of our method in BFS and DFS partitioning scheme is generally higher than that in random. For the SHS27k dataset, our method achieves an absolute improvement of $1.43 \%$, $10.75 \%$, $3.48 \%$ when compared with the GNN-PPI model in random, BFS, and DFS partitioning methods, respectively. And for the SHS148k dataset, our method achieves an absolute improvement of $0.12 \%$, $2.61 \%$, $1.12 \%$ when compared with the GNN-PPI method in random, BFS, and DFS partitioning methods, respectively. This illustrates that our method has a certain generalization ability and has practical implications.

**Table 1:** Performance results of LDMGNN compared with other methods on two datasets, we report the mean and standard deviation of the test sets

Methods	SHS27k			SHS148k		
	Random	BFS	DFS	Random	BFS	DFS
SVM [33]	$75.35 \pm 1.05$	$42.98 \pm 6.15$	$53.07 \pm 5.16$	$80.55 \pm 0.23$	$49.14 \pm 5.30$	$58.59 \pm 0.07$
RF [34]	$78.45 \pm 0.08$	$37.67 \pm 1.57$	$35.55 \pm 2.22$	$82.10 \pm 0.20$	$38.96 \pm 1.94$	$43.26 \pm 3.43$
LR [35]	$71.55 \pm 0.93$	$43.06 \pm 5.05$	$48.51 \pm 1.87$	$67.00 \pm 0.07$	$47.45 \pm 1.42$	$51.09 \pm 2.09$
HIN2Vec [37]	$74.22 \pm 2.38$	$49.61 \pm 4.88$	$53.78 \pm 3.05$	$78.01 \pm 0.62$	$56.94 \pm 3.20$	$57.15 \pm 2.49$
SDNE [38]	$84.04 \pm 0.91$	$47.29 \pm 4.32$	$53.42 \pm 2.82$	$86.65 \pm 2.73$	$58.43 \pm 4.94$	$68.84 \pm 1.52$
LPI-DLDN [39]	$77.36 \pm 0.48$	$44.68 \pm 2.31$	$54.98 \pm 3.94$	$83.83 \pm 0.52$	$56.41 \pm 5.38$	$60.07 \pm 2.71$
LPI-deepGBDT [40]	$72.70 \pm 0.67$	$42.25 \pm 3.81$	$50.48 \pm 2.76$	$81.69 \pm 0.39$	$55.51 \pm 7.40$	$59.67 \pm 3.29$
DTI-CDF [41]	$79.29 \pm 0.89$	$49.60 \pm 5.28$	$55.88 \pm 4.19$	$83.12 \pm 0.55$	$60.04 \pm 8.27$	$65.42 \pm 5.89$
PIPR [7]	$83.31 \pm 0.75$	$44.48 \pm 4.44$	$57.80 \pm 3.24$	$90.05 \pm 2.59$	$61.83 \pm 10.23$	$63.98 \pm 0.76$
GAT [42]	$86.35 \pm 0.86$	$53.08 \pm 5.24$	$60.09 \pm 1.69$	$88.87 \pm 0.31$	$62.10 \pm 7.75$	$65.49 \pm 0.50$
GNN-PPI [26]	$87.91 \pm 0.39$	$63.81 \pm 1.79$	$74.72 \pm 5.26$	$92.26 \pm 0.10$	$71.37 \pm 5.33$	$82.67 \pm 0.85$
**LDMGNN**	**89.34** $\pm \; 0.44$	**74.56** $\pm \;3.03$	**78.20** $\pm \; 2.69$	**92.38** $\pm \;0.08$	**73.98** $\pm \;5.51$	**83.79** $\pm \; 0.95$

Each boldface number represents the best value for that partition

### Significant difference analysis

To verify whether the performance of our proposed LDMGNN model is statistically significantly different from these 11 baseline models, we conducted a paired samples t-test using SPSS software. The related results are shown in Tables 2 and 3. Table 2 is used to represent the correlation between two samples, where the value of correlations is in the interval [$-1$,1]. When this value is greater than 0, it means that there is a positive correlation between the two samples, and when this value is less than 0, it means that there is a negative correlation between the two samples. And the larger the absolute value of the correlations, the stronger the correlation between the two samples. At the same time, the significance level *p* value (i.e., Sig. in Table 2) should be less than 0.05. When the *p* value is less than 0.05, it can indicate whether the correlation between samples is significant. A paired samples t-test only makes sense when there is a significant correlation between paired samples. Table 3 shows the results of the paired samples t-test, when the *p* value (i.e., Sig.(2-tailed)) is less than 0.05, it indicates that there is a significant difference between the two samples.

As shown in Table 2 , the correlation of the first row (LDMGNN & SVM) is 0.976 (the absolute value is close to 1), and the significance level *p* value (Sig.) is 0.001 (< 0.05), which indicates that the two samples of LDMGNN and SVM have significant correlation, and is strongly correlated. Similarly, it can be seen that the correlations of the last 10 pairs of samples are 0.917, 0.920, 0.916, 0.953, 0.938, 0.929, 0.943, 0.916, 0.938 and 0.965, respectively (all greater than 0.9). And the corresponding significance level *p* value (i.e., Sig.) of the last 10 paired samples are 0.010, 0.009, 0.010, 0.003, 0.006, 0.007, 0.005, 0.010, 0.006 and 0.002 (all less than 0.05). Obviously, these 10 paired samples are all significantly correlated. Therefore, it is meaningful to perform a paired samples t-test on these 11 pairs of paired samples.

**Table 2:** Paired samples correlation of the LDMGNN model with 11 baseline models

		N	Correlation	Sig.
Pair 1	LDMGNN &SVM	6	0.976	0.001
Pair 2	LDMGNN &RF	6	0.917	0.010
Pair 3	LDMGNN &LR	6	0.920	0.009
Pair 4	LDMGNN &HIN2Vec	6	0.916	0.010
Pair 5	LDMGNN &SDNE	6	0.953	0.003
Pair 6	LDMGNN &LPIDLDN	6	0.938	0.006
Pair 7	LDMGNN &LPIdeepGBDT	6	0.929	0.007
Pair 8	LDMGNN &DTICDF	6	0.943	0.005
Pair 9	LDMGNN &PIPR	6	0.916	0.010
Pair 10	LDMGNN &GAT	6	0.938	0.006
Pair 11	LDMGNN &GNNPPI	6	0.965	0.002

**Table 3:** Paired samples test of the LDMGNN model with 11 baseline models

					95% confidence interval of the difference				
		Mean	SD	Std. error mean	Lower	Upper	t	df	Sig.(2-tailed)
Pair 1	LDMGNN-SVM	22.09500	7.58174	3.09523	14.13845	30.05155	7.138	5	**0.001**
Pair 2	LDMGNN-RF	29.37667	14.80078	6.04239	13.84420	44.90913	4.862	5	**0.005**
Pair 3	LDMGNN-LR	27.26500	5.42631	2.21528	21.57044	32.95956	12.308	5	**0.000**
Pair 4	LDMGNN-HIN2Vec	20.42333	5.50138	2.24593	14.64998	26.19668	9.093	5	**0.000**
Pair 5	LDMGNN-SDNE	15.59667	9.21296	3.76118	5.92826	25.26507	4.147	5	**0.009**
Pair 6	LDMGNN-LPIDLDN	19.15333	7.98559	3.26010	10.77297	27.53369	5.875	5	**0.002**
Pair 7	LDMGNN-LPIdeepGBDT	21.65833	7.89829	3.22446	13.36958	29.94708	6.717	5	**0.001**
Pair 8	LDMGNN-DTICDF	16.48333	6.47322	2.64268	9.69010	23.27656	6.237	5	**0.001**
Pair 9	LDMGNN-PIPR	15.13333	10.28307	4.19805	4.34191	25.92476	3.605	5	**0.015**
Pair 10	LDMGNN-GAT	12.71167	7.96402	3.25130	4.35394	21.06940	3.910	5	**0.011**
Pair 11	LDMGNN-GNNPPI	3.25167	3.85639	1.57436	-0.79537	7.29870	2.065	5	**0.094**

Bold number represents the *p*-value of the corresponding paired sample

From the Table 3, we can see that the *p* values (i.e., Sig.(2-tailed)) of the top 10 pairs of samples are 0.001, 0.005, 0.000, 0.000, 0.009, 0.002, 0.001, 0.002, 0.015 and 0.011, respectively, all of which are less than 0.05. It shows that there are significant differences between the first 10 pairs of samples. The *p* values (i.e., Sig.(2-tailed)) of the 11th pair of samples is 0.094, which is greater than 0.05. As for the 11th pair of samples, we consider that the extreme imbalance of the data may be a factor in this situation. Further, from the perspective of reality, we believe that LDMGNN model has certain practical significance. Lv et al. [26] studied the Homo sapiens subsets at two time points (2011 / 01 / 25 and 2021 / 01 / 25) in the BioGRID database. They found that the newly discovered proteins had local patterns of BFS and DFS. In these two partition schemes, our LDMGNN model has a large improvement in accuracy compared with the GNN-PPI model.

### Ablation analysis

In order to verify the importance and effectiveness of each module in this study for the prediction model, we conduct an ablation study by deleting or replacing each module in this study. We use GNN-PPI as the baseline for PPIs prediction, which processes amino acid sequences using RNN and aggregates only first-order neighbor information. −PMHGE represents the removal of PMHGE from the LDMGNN model and, unlike baseline GNN-PPI, and uses the Transformer with a multi-head self-attention mechanism to learn the amino acid interdependency in the sequence. -PAASE represents the deletion of the PAASE module from the LDMGNN model. Unlike the baseline GNN-PPI, we construct a THPPI network and simultaneously aggregates first-order and second-order neighbor information, increasing the spatial receptive field in the model. LDMGNN is our proposed model. Compared with the baseline GNN-PPI, our LDMGNN not only captures the long-distance dependency information in the sequence but also increases the spatial receptive field in space. We still use Micro-F1 as the evaluation metric.

**Table 4:** Ablation studies on SHS27k and SHS148k datasets

Datasets	Partition scheme	GNN-PPI	−PMHGE	−PAASE	LDMGNN
SHS27K	Random	$87.91 \pm 0.39$	$88.72 \pm 0.44$	$89.28 \pm 0.41$	$89.34 \pm 0.44$
	BFS	$63.81 \pm 1.79$	$68.84 \pm 5.49$	$68.61 \pm 3.72$	$74.56 \pm 3.03$
	DFS	$74.72 \pm 5.26$	$76.92 \pm 4.28$	$74.81 \pm 2.78$	$78.20 \pm 2.69$
SHS148K	Random	$92.26 \pm 0.10$	$92.38 \pm 0.08$	$92.35 \pm 0.26$	$92.54 \pm 0.21$
	BFS	$71.37 \pm 5.33$	$69.01 \pm 4.36$	$71.63 \pm 3.19$	$73.98 \pm 5.51$
	DFS	$82.67 \pm 0.85$	$82.77 \pm 1.58$	$82.91 \pm 1.19$	$83.79 \pm 0.95$

As can be seen from Table 4, when the model only uses the multi-head self-attention mechanism to capture the long-distance dependency information in the sequence, for the SHS27k dataset, the current model increases by $0.81 \%$, $5.03 \%$ and $2.20 \%$ respectively compared with the GNN-PPI model under the random, BFS and DFS partitioning schemes. For the SHS148k dataset, $0.12 \%$, $-2.36 \%$ and $0.10 \%$ improvements are achieved respectively. The results show that the prediction accuracy could be improved if the model only captures the interdependency of amino acids in the sequence, indicating that the long-distance dependency of amino acids in the sequence plays a positive role in the prediction of multi-label PPI. In view of the situation that micro-F1 of the SHS148k dataset decreased by $2.36 \%$ in the BFS partitioning scheme, we believe that this is caused by data imbalance.

When the model only increases the spatial receptive field of network, for the SHS27k dataset, the current model increases by $1.37 \%$, $4.80 \%$ and $0.09 \%$ respectively compared with the GNN-PPI model under the random, BFS and DFS partitioning schemes. For the SHS148k dataset, $0.09 \%$, $0.26 \%$ and $0.24 \%$ improvements are achieved respectively. The results show that aggregating the information of first-order and second-order neighbors simultaneously can improve the accuracy of prediction, which suggest that appropriate increase of network receptive field also plays a positive role in the prediction of multi-label PPI. However, the improved accuracy is the highest when the model captures the interdependencies between amino acids in the sequence and aggregate the first-order and second-order neighbors, which further demonstrate that LDMGNN model is effective for predicting multi-label PPI.

### The selection of hop number

When constructing a multi-hop PPI network to increase the receptive field, we conducted experiments on different *k* to determine the *k*-hop network to be constructed. The experimental results are shown in Table 5. In the table, “One-Hop” indicates the case where $k = 1$. In this case, the PPI network is the original PPI network, and the target node only aggregates the information of first-order neighbors. In the table, “One-Hop $+$ Two-Hop” represents the case where $k=2$. We construct a THPPI network, where the target node aggregates the information of first-order and second-order neighbors simultaneously. “One-Hop $+$ Two-Hop $+$ Three-Hop” in the table represents $k=3$. We construct both a THPPI network and a Three-Hop PPI network, and the target node aggregates the information of first-order, second-order, and third-order neighbors at the same time. We still use micro-F1 as the evaluation metric, and each boldface number means the best accuracy under this partitioning scheme. Obviously, the prediction accuracy is the highest when *k* is 2.

**Table 5:** Comparison experiment of hop number selection on SHS27k and SHS128k datasets

Datasets	Partition scheme	One-hop	One-hop $+$ two-hop	One-hop $+$ two-hop $+$ three-hop
SHS27K	Random	$88.72 \pm 0.44$	**89.34** $\pm \; 0.44$	$88.24 \pm 0.95$
	BFS	$68.84 \pm 5.49$	**74.56** $\pm \; 3.03$	$68.42 \pm 6.40$
	DFS	$76.92 \pm 4.28$	**78.20** $\pm \;2.69$	$74.52 \pm 4.33$
SHS148K	Random	$92.38 \pm 0.08$	**92.54** $\pm \; 0.21$	$92.28 \pm 0.13$
	BFS	$70.92 \pm 5.37$	**73.98** $\pm \; 5.51$	$69.01 \pm 4.36$
	DFS	$82.77 \pm 1.58$	**83.79** $\pm \;0.95$	$81.96 \pm 0.93$

Bold number represents the best value for that partition scheme

It can be seen from Table 5 that when *k* is set to 2, the accuracy of the model obtained in each partition scheme on the two datasets is higher than that when *K* is set to 1. This indicates that it is necessary to construct a multi-hop PPI network to increase the receptive field of the network in space. However, with the increase of *k*, the performance of the model on the two datasets shows a decreasing trend. When *k* is 3, the model acquires much less accuracy in each of the partition methods on both datasets than when *k* is 2. Indeed, the accuracy obtained when *k* is 3 is less in all cases than when *k* is 1. This indicates that the simple construction of a multi-hop PPI network is not the best. As the receptive field gradually increases, the model gradually tends to be over-fitting. Therefore, in this study, *k* is selected as 2 when we construct the multi-hop PPI network. It further shows that it is effective and reasonable for us to aggregate the first-order and second-order neighbor information at the same time and appropriately increase the receptive field of the network.

### The effect of more negative samples on model performance

To test how more negative samples will affect the performance of the model, we increase the number of negative samples while keeping the number of positive samples constant. At this time, the positive: negative sample rate in the datasets will change. Specifically, for the SHS27k dataset, we randomly selected three negative samples from proteins pairs with unknown interactions, and the number of these three negative samples were 22,872, 38,120 and 76,240, respectively. We took the protein pairs that are known interactions as positive samples, and the number is 7624. Thus we can obtain three different positive:negative sample rates, which are 1:3, 1:5 and 1:10. Similarly, for the SHS148k dataset, we randomly selected three negative samples from protein pairs of unknown interactions, with numbers of 133,464, 222,440, and 444,880, respectively. We also took known interacting protein pairs as positive samples with a number of 44,488, resulting in three different positive:negative sample rates, which are 1 : 3, 1 : 5, and 1 : 10, respectively. We still choose three partition schemes to divide the test set, namely random, BFS and DFS, and our test set accounts for $20 \%$ of the dataset.

The experimental results are shown in Table 6, where 1:1 is the positive:negative sample rate used by the LDMGNN model. As can be seen from Table 6, for the SHS27k dataset, under random, BFS and DFS partition schemes. Compared with the result that positive:negative sample rate is 1:1, when the positive:negative sample rate is 1:3, the performance of the model decreases by 3.09, 3.13 and 2.06, respectively; when the rate is 1:5, the performance of the model decreases by 6.60, 5.27 and 5.26, respectively; when the rate is 1:10, the performance of the model decreases by 11.40, 8.94 and 8.36, respectively. And for the SHS148k dataset, under random, BFS and DFS partition schemes. Compared with the result that positive:negative sample rate is 1:1, when the positive:negative sample rate is 1:3, the performance of the model decreases by 3.07, 2.26 and 4.82, respectively; when the rate is 1:5, it decreases by 10.28, 5.77 and 11.15, respectively; when the rate is 1:10, it decreases by 16.83, 12.62 and 16.87, respectively.

Obviously, we can see that when we add more negative samples, the performance of the model will decrease significantly. This is because when the number of negative samples exceeds the number of positive samples, it will affect the correct judgment of the model on the positive samples, so that the classifier can not capture the features of the positive samples well. Therefore, the imbalance between positive and negative samples will negatively affect the performance of the model.

**Table 6:** Results of increase the number of negative samples on SHS27K and SHS148k

Positive:negative sample rate	SHS27k			SHS148k		
	Random	BFS	DFS	Random	BFS	DFS
1:1	**89.34** $\pm \; 0.44$	**74.56** $\pm \; 3.03$	**78.20** $\pm \;2.69$	**92.38** $\pm \; 0.08$	**73.98** $\pm \; 5.51$	**83.79** $\pm \; 0.95$
1:3	$86.25 \pm 0.21$	$71.43 \pm 1.56$	$76.14 \pm 1.66$	$89.31 \pm 0.98$	$71.72 \pm 2.34$	$78.97 \pm 0.75$
1:5	$82.74 \pm 0.55$	$69.29 \pm 2.71$	$72.94 \pm 2.81$	$82.10 \pm 2.39$	$68.21 \pm 4.84$	$72.64 \pm 0.93$
1:10	$77.94 \pm 0.75$	$65.62 \pm 2.47$	$69.84 \pm 1.28$	$75.55 \pm 0.52$	$61.36 \pm 5.41$	$66.92 \pm 1.85$

Bold number represents the best value for that partition scheme

## Discussion

Next, we will introduce why we use the Transformer with a multi-head self-attention mechanism to capture amino acid sequence information and why we construct a THPPI network to aggregate two-hop neighbor information.

It is acknowledged that the Transformer [43] was first proposed to replace recurrent neural network (RNN) to solve natural language processing. It has two unique properties, one is that it can obtain the long-distance dependency of sequences, and the other is that it can be parallelized. Inspired by the methods of natural language processing such as Bert [44] and Roberta [45], we regard each amino acid as a vector, the amino acid sequence as a vector set. And we consider that there may also be long-distance dependency between every two amino acids in a sequence. So we use the Transformer with a multi-head self-attention mechanism to capture amino acid sequence information.

**Fig. 1:**
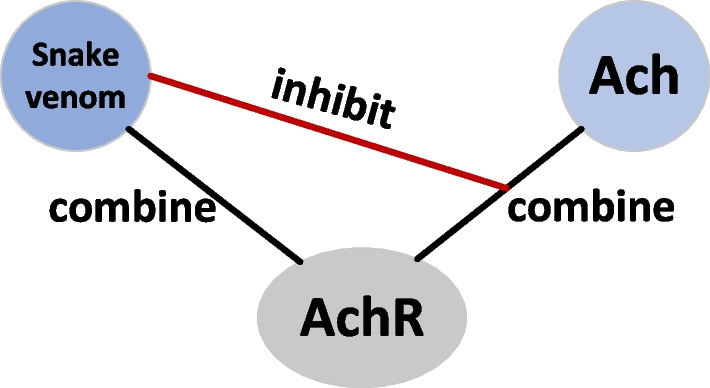
The illustration of the competitive inhibition

Meanwhile, we constrcut a THPPI network since there may be meaningful information between two indirectly interacting nodes. Exactly, the competitive inhibition [46] in biochemistry can also explain that there may be some structural similarity or functional similarity between the two indirectly connected nodes. As shown in Fig. 1, a typical example [47] of similar structures in biomolecules causing competitive inhibition. When humans are bitten by snakes, snake venom proteins follow the blood circulation into the nervous tissue space, bind to acetylcholine receptors (AchR), and the binding affinity between them is much higher than that of acetylcholine (Ach). Thus the snake venom proteins would inhibit the binding of acetylcholine to acetylcholine receptors. Here, the snake venom proteins are structurally similar to acetylcholine. Enlightened by this case, we consider that in a PPI network, there may be two proteins that are indirectly connected, but structurally similar or functionally similar. So we construct a THPPI network to aggregate the second-order neighbor information to enlarge the receptive field in space.

## Conclusions

In this study, we propose the LDMGNN model to predict multi-label protein–protein interactions. LDMGNN first captures the potential features of amino acid sequences through the PAASE module and then concatenates this information with the topological information of the initial PPI network and the topological information of the THPPI network respectively. Then, they are respectively input into the graph neural network, and the two obtained feature matrices are addition by element-wise as the final embedding of protein and protein pair. Finally, this embedding is fed into a classifier for predicting protein–protein interactions.

We carry out a series of experiments, and in the SHS27k dataset and SHS148k dataset, our model shows better performance than the existing model. Furthermore, we perform an ablation experiment to verify that each module in the model is indispensable and that the parameters in the experiment are reasonable and effective. This indicates that the Transformer with a multi-head self-attention mechanism can successfully capture the long-distance dependence information in amino acid sequences. The spatial receptive field of the network can be increased by aggregating the first-order and second-order neighbor information simultaneously. In conclusion, the LDMGNN model can comprehensively learn the feature information between protein pairs and has a good potential in PPI prediction.

## Methods

This section introduces the proposed multi-label PPI prediction approach, which is an end-to-end representation learning model. Given the representation of protein amino acid sequence, the adjacency matrix of PPI network and the adjacency matrix of THPPI network, we try to predict the labels between protein–protein pairs. The representation of protein amino acid sequence input here is processed into a numerical vector by [7]. In this section, we define the multi-label PPI prediction problem. Then we will introduce our LDMGNN model in detail.

### Problem formulation

We represent the set of amino acids as *M*, and define the amino acid sequence *S* of a protein, which consists of amino acids in varying proportions as $S=\left\{ m_{1}, m_{2}, \ldots , m_{l}\right\}$, where $m_{i} \in M, i=1,2, \ldots , l$. We consider the initial PPI network as an undirected graph $G_{1}=\langle {P}, {I}\rangle$, whose adjacency matrix is $A_{1} \in \{0,1\}^{N \times N}$, where *P* is the set of proteins and denoted as $P=\left\{ p_{1}, p_{2}, \ldots , p_{n}\right\}$. *I* is the set of protein–protein interactions, defined as $I=\left\{ p_{i j} \mid p_{i j}=\left( p_{i}, p_{j}\right) , i \ne j, p_{i}, p_{j} \in P\right\}$. If $p_{ij}=1$, this indicates that there have interactions between protein $p_{i}$ and protein $p_{j}$. If $p_{ij}=0$, this indicates that there is no interaction between the proteins or the interactions between them has not been identified at this time. Similarly, we consider the constructed THPPI network as an undirected graph $G_{2}=\langle {P}, {I}\rangle$, whose adjacency matrix is $A_{2} \in \{0,1\}^{N \times N}$.

The task of this multi-label classification is to learn a model $F=(p, {\hat{y}})$ from the training set $I_{train}$, and its input *p* is protein pairs with known interaction, $p \in \ I_{train}$. The output is a 7-dimensional vector, which corresponds to a finite set of labels *L*. We define the label set of PPIs as $L=\left\{ \ell _{0}, \mathrm {\ell _{1}, \ldots , \ell }_{n}\right\}$, where $n=6$ are the types of protein–protein interaction, which are post-translational modifications (PTMOD), catalysis, reaction, activation, expression, binding and inhibition, respectively. The interaction of each pair of proteins contains at least one type. When there is a certain type of interaction, the corresponding position in the vector is 1, otherwise it is 0. The learned model *F* is used to predict the labels ${\hat{y}}_{ij}$ of protein pair $p_{ij} \in \ I_{test}$.

### Overview

**Fig. 2:**
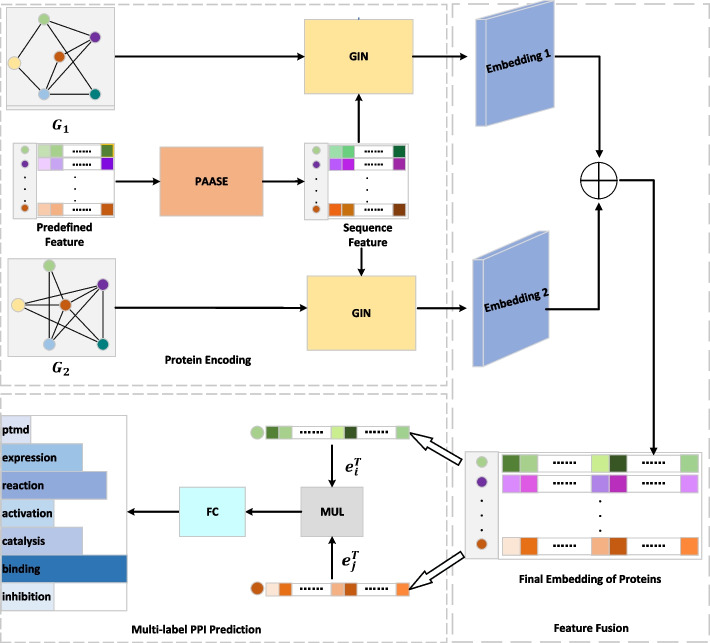
The illustration of the LDMGNN framework. Here $G_{1}$ indicates the original PPI network, and $G_{2}$ indicates the THPPI network. This symbol $\oplus$ indicates addition by element-wise. $e_i^{T}$ and $e_j^{T}$ represent embedding vectors for proteins $p_i$ and $p_j$, respectively. And the MUL block represents the dot product operation. The FC block is designed to classify the classes of protein interactions. And as shown in the figure, there are seven different types

The framework of the proposed LDMGNN model is shown in Fig. 2. We introduce the framework from the following three parts. The first part is the “Protein Encoding”, which is the core of LDMGNN model. It is used to extract the representation of protein nodes. The second part is “Feature Fusion”, and the last part is the “Multi-label PPI Prediction”.

### Protein encoding

In the process of protein encoding, we can regard this process as two parts. These two parts are trained together in an end-to-end manner. One is protein amino acid sequence encoding (PAASE), which is to capture the protein feature based on amino acid sequence, which we call sequence feature. The second is protein multi-hop graph encoding (PMHGE), which can be regarded as composed of two branches. One branch uses a GIN block to capture the first embedding of protein, and the other branch uses a GIN block to capture the second embedding of protein.

### Protein amino acid sequence encoding (PAASE)

This module is used to capture the protein feature based on amino acid sequence. Predefined feature appearing in modules were observed by [7] through processing. Chen et al. [7] used the embedding method to represent each amino acid $m \in M$ as a 13 dimensional vector, which is composed of two sub-vectors, i.e., $m=\left[ m_{1}, m_{2}\right]$. The first sub-vector $m_1$ measures the co-occurrence similarity of amino acids, and its dimension is 5. The second sub-vector $m_2$ represents the similarity of the electrostatic and hydrophobic among amino acids, which is an eight-dimensional one-hot encoding.

**Fig. 3:**
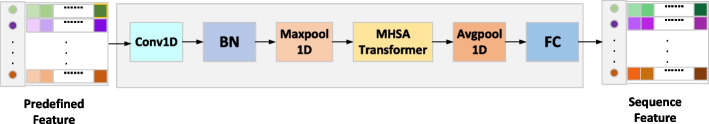
The illustration of PAASE module. The MHSA Transformer block here refers to the Transformer with the multi-head self-attention mechanism, just for the convenience of writing in the figure, so the multi-head self-attention is replaced by MHSA

As shown in Fig. 3, the predefined feature are input and pass through a one-dimensional convolution layer, and the size of the convolution kernel is 3. We input the hidden features of the output into the normalization layer, which can increase the learning rate of the model and speed up the training speed. Then we choose a maximum pooling layer and let it extract more representative features. To capture the long-distance dependency information in the sequence, we then input these representative features into the Transformer module with multi-head self-attention mechanism for learning about the interdependencies of amino acids. Next, We input the hidden features obtained from the MHSA Transformer layer into a one-dimensional average pooling layer, for which dimension reduction will be performed. Finally, the sequence feature is obtained through a fully connected layer.

#### The transformer with multi-head self-attention mechanism

The function of this block is to learn the interdependence between each two amino acids in the amino acid sequence by calculating the correlation coefficient between them. This can not only capture the local information of amino acids in the sequence, but also capture the long-distance dependency information between amino acids.

**Fig. 4:**
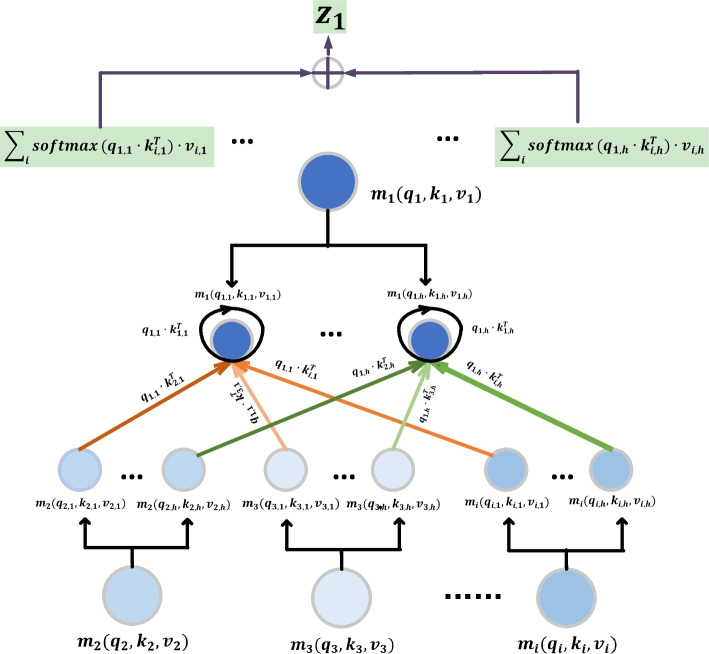
The illustration of the multi-head self-attention mechanism. The multi-head self-attention mechanism operates on each amino acid node. $m_{i}\left( q_{i}, k_{i}, v_{i}\right)$ represents the query vector, key vector and value vector of amino acid node $m_{i}$. $m_{i}\left( q_{i, h}, k_{i, h}, v_{i, j}\right)$ represents the query vector, key vector, and value vector of the *h*th type of amino acid node $m_{i}$. This symbol $\oplus$ indicates addition by element-wise

Because of the potential for multiple types of interactions between each pair of amino acids, we apply multi-head self-attention mechanism to each amino acid of each protein amino acid sequence. Then we extract the low dimensional feature embedding of each amino acid by computing the correlation of different kinds between each pair of amino acids. As shown in Fig. 4, and for convenience, we only present a brief diagram. For each amino acid $m_{i}$, it gets a query vector $q_{i} \in R^{dq}$, a key vector $k_{i} \in R^{dk}$, and a value vector $v_{i} \in R^{dv}$. They are obtained by linear transformations of the features of amino acids using trainable parameters $W_{q} \in R^{\text {feature} \_ \text {in} \times {dq}}$, $W_{k} \in R^{\text {feature} \_ \text {in} \times {dk}}$ and $W_{v} \in R^{\text {feature} \_ \text {in} \times {dv}}$, which are shared for all amino acid nodes.

Since there are *h* types of correlations for each pair of amino acids $\left( m_{i}, m_{j}\right)$, it is necessary to calculate the embeddings of amino acid nodes using the multi-head self-attention mechanism. For each node $m_{i}$ in the amino acid sequence, $q_{i, 1}, \ldots , q_{i, h}$ can be obtained by linear transformation of $q_{i}$ using different weight matrices $W_{q, 1}, \ldots , W_{q, h}$. Smilarly, $k_{i, 1}, \ldots , k_{i, h}$ can be obtained by linear transformation of $k_{i}$ using different weight matrices $W_{k, 1}, \ldots , W_{k, h}$; $v_{i, 1}, \ldots , v_{i, h}$ can be obtained by linear transformation of $v_{i}$ using different weight matrices $W_{v, 1}, \ldots , W_{v, h}$. For each correlation in each pair of amino acids, its coefficients $\alpha _{i, j}^{h}$ can be calculated using the Query-key dot product method, which can be expressed by mathematical formula as follows:

4 $$\begin{aligned} \alpha _{i, j}^{h}=q_{i, h} \ldots k_{j, h}^{T}. \end{aligned}$$

These correlation coefficients will then be normalized. The correlation coefficient $\alpha _{i, j}^{h}$ is used as the weight to measure the value of each amino acid $m_{i}$, and then the weighted sum is carried out to obtain the embedding of *h* types of each amino acid $m_{i}$, which are $z_{i}^{1}, \ldots , {z}_{i}^{h}$, respectively. Finally, these embeddings of all kinds are concatenated with the Transformer to produce the final output $z_{i}$ of an amino acid node in the current layer, which is expressed by mathematical formula as:

5 $$\begin{aligned} Z_{i}={\text {concat}}\left( z_{i}^{1} \ldots z_{i}^{h}\right) \cdot W_{o} , \end{aligned}$$

where $$Z_{i} \in R^{\text{ feature }\_\text {out }}$$, and

6 $$\begin{aligned} z_{i}^{h}=\sum _{j} {\text {softmax}}_{j}\left( \frac{\alpha _{i, j}^{h}}{\sqrt{d_{k}}}\right) \cdot v_{j}^{h}. \end{aligned}$$

### Protein multi-hop graph encoding (PMHGE)

In the process of protein multi-hop graph encoding, we use two GIN blocks to obtain two embeddings of protein. The input of the first GIN block is the original PPI network and the sequence feature, and the output is the first embedding $E_1$ of protein. The input of the second GIN block is the two-hop PPI network (THPPI) and the sequence feature, and output is the second embedding $E_2$ of protein. In this way, not only the features of adjacent nodes can be aggregated directly, but also the features of non adjacent nodes can be aggregated.

#### Two-hop protein–protein interaction (THPPI) network

Through the THPPI graph network, the GIN block can learn new interactions between two proteins for the purpose of augmented graph representation. We construct the THPPI network through the PPI network. Specifically, we generate the adjacency matrix $A^{2}$ of the THPPI network $G_{2}$ through the adjacency matrix *A* of the original graph $G_{1}$, so as to obtain the structural information of the THPPI network. The mathematical formula is described as follows:

7 $$\begin{aligned} A^{2}={\text {sign}}\left( A \cdot A^{T}\right) , \end{aligned}$$

where ${\text {sign}}(x)$ is a symbolic function, when $x>0$, the value is 1; and when $x \le 0$, the value is 0. It is worth noting that the new adjacency matrix contains the self-connection relation. And when protein nodes *i* and *j* correspond to values greater than 0, indicating that there is two-hop relationship between them on the original graph. Similarly, in the THPPI network $G_{2}$, two protein nodes are connected by edges to indicate their interaction. Due to the nature of graphs in general, the model of graph neural networks can also do message passing and aggregation operational on $G_{2}$.

#### Graph isomorphic network (GIN) block

**Fig. 5:**
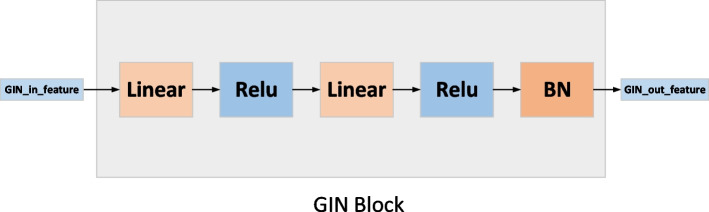
The illustration of GIN block

The two GIN blocks in Fig. 2 are the same, as shown in Fig. 5 below. The input of this module is an $L^{*} 256$ feature matrix, where *L* represents the number of proteins and 256 represents the number of features of each protein. This feature matrix is obtained by concatenating structural information of the PPI network with amino acid sequence information. After two linear layers, two ReLU activation layers and normalization layers, the protein embedding was obtained.

Graph neural networks [24, 48–50] have seen tremendous progress in a variety of extremely challenging tasks. While graph isomorphism network (GIN) [51] is proved to be the most powerful variant of graph neural network (GNN) at present. Next, we will introduce how to obtain the information of the PPI network and the information of the THPPI network through the GIN block.

Similar to graph neural networks, the neighbor aggregation mechanism is the core of GIN. We iteratively update the feature of each node by aggregating feature of its neighbors. After *k* iterations, the structure information in the *k*-hop neighborhood can be captured. And the new feature vector $g_{p}^{k}$ of node *p* can be expressed by the following mathematical formula:

8 $$\begin{aligned} g_{p}^{k}={\text {Update}}\left( \left\{ g_{p}^{k-1}, a_{p}^{k}\right\} \right) , a_{p}^{k}={\text {Agg}}\left( \left\{ g_{p^{\prime }}^{k-1} \mid p^{\prime } \in N(p)\right\} \right) . \end{aligned}$$

where *N*(*p*) is the set of all neighbor nodes of node *p*. We choose vector sum as the aggregation function and multi-layer perceptrons (MLP) as the update function.

Then, for the original PPI network $G_{1}$, after *k*th iterations, node *p* obtains the feature vector $g_{p_{1}}^{k}$, which can be expressed as:

9 $$\begin{aligned} g_{p_{1}}^{k}=M L P^{k}\left( \left( 1+\epsilon _{1}^{k}\right) +\sum _{p_{1}^{\prime } \in N(p)} g_{p_{1}^{\prime }}^{k-1}\right) , \end{aligned}$$

where $p_{1}^{\prime }$ represents the first-order neighbor of the node *p*, and $\epsilon _{1}$ is hyperparameter. Finally, we can obtain the embedding of $G_1$, which is called $E_1$.

Similarly, for the THPPI network $G_{2}$, after *k*th iterations, node *p* obtains the feature vector $g_{p_{2}}^{k}$, which can be expressed as:

10 $$\begin{aligned} g_{p_{2}}^{k}=M L P^{k}\left( \left( 1+\epsilon _{2}^{k}\right) +\sum _{p_{2}^{\prime \prime } \in N(p)} g_{p_{2}^{\prime \prime }}^{k-1}\right) , \end{aligned}$$

where $p_{2}^{\prime \prime }$ represents the second-order neighbor of node *p*, and $\epsilon _{2}$ is hyperparameter. On the original graph $G_1$, $p_{2}^{\prime \prime }$ is the second-order neighbor of *p*. However, on the graph $G_2$ we constructed, $p_{2}^{\prime \prime }$ is the first-order neighbor of *p*. Finally, we can obtain the embedding of $G_2$, which is called $E_2$.

### Feature fusion

This operation can well integrate the embedding $E_1$ of the original PPI network and the embedding $E_2$ of the THPPI network into the same embedding space. We fuse these two embeddings together to obtain the final embedding $E_{\text{ out } }$ of all proteins and use element-wise summation as the fusion form in this paper. Expressed in mathematical formula as the following:

11 $$\begin{aligned} E_{\text{ out } } =E_1 + E_2. \end{aligned}$$

### Multi-label PPI prediction

We input the final embedding $E_{\text{ out } }$ of the proteins into a fully connected (FC) layer classifier, which predicts the interactions between two proteins. We use dot product operation to combine the embedding $e_i$ of protein $p_i$ and the embedding $e_j$ of protein $p_j$. The mathematical formula is as follows:

12 $$\begin{aligned} {\hat{y}}_{i j}=FC\left( e_i \cdot e_j\right) . \end{aligned}$$

In order to better supervise the training process of the model, we choose the multi-task binary cross-entropy loss function. And its mathematical formula is shown as follows:

13 $$\begin{aligned} L=\sum _{k=0}^{n}\left( \sum _{p_{i j} \in {\mathcal {I}}_{\text{ train } }}-y_{i j}^{k} \log {\hat{y}}_{i j}^{k}-\left( 1-y_{i j}^{k}\right) \log \left( 1-{\hat{y}}_{i j}^{k}\right) \right) , \end{aligned}$$

where ${\mathcal {I}}_{\text{ train } }$ represents the training set. $y_{i j}^{k}$ and ${\hat{y}}_{i j}^{k}$ denotes the ground-truth label and predicted probability for class *k*, respectively.

## Data Availability

The datasets used in this study can be downloaded from the http://yellowstone.cs.ucla.edu/~muhao/pipr/SHS_ppi_beta.zip or from the https://drive.google.com/open?id=1y_5gje6AofqjrkMPY58XUdKgDuu1mZCh. The source code is available online at: https://github.com/666Coco123/LDMGNN.

## Abbreviations

PPI: Protein–protein interaction
THPPI: Two-hop protein–protein interaction
DNA: Deoxyribonucleic acid
Y2H: Yeast two-hybrid
MS-PCI: Mass spectrometric protein complex identification
TAP: Tandem affinity purification
SVM: Support vector machine
RF: Random forest
LR: Logistic regression
SAE: Stacked auto encoder
GCN: Graph convolutional networks
GIN: Graph isomorphism networks
RNN: Recurrent neural networks
ER: Erdös–Rényi
DFS: Depth first search
BFS: Breadth first search
AchR: Acetylcholine receptors
Ach: Acetylcholine
PAASE: Protein amino acid sequence encoding
MHSA: Multi-head self-attention
PMHGE: Protein multi-hop graph encoding
LDMGNN: Long-distance dependency combined multi-hop graph neural networks
